# Systolic Blood Pressure and Longitudinal Progression of Arterial Stiffness: A Quantitative Meta‐Analysis

**DOI:** 10.1161/JAHA.120.017804

**Published:** 2020-08-28

**Authors:** Jack Wilson, Alastair John Stewart Webb

**Affiliations:** ^1^ Wolfson Centre for Prevention of Stroke and Dementia University of Oxford United Kingdom

**Keywords:** arterial stiffness, hypertension, longitudinal cohort study, meta‐analysis, systematic review, Hypertension, Epidemiology

## Abstract

**Background:**

Arterial stiffness predicts the risk of cardiovascular events and all‐cause mortality and is associated with age and hypertension. However, the magnitude of the relationship between blood pressure (BP) and progression of arterial stiffness is unclear, limiting our understanding of how arterial stiffness mediates clinical effects of hypertension and planning of clinical trials.

**Methods and Results:**

Medline and EMBASE were searched for prospective studies reporting linear models between baseline BP and progression of arterial stiffness, with and without adjustment for demographic characteristics and baseline stiffness. Standardized and unstandardized β coefficients for pulse wave velocity were combined by fixed and random effects meta‐analysis, weighted by the inverse variance. Of 566 fully reviewed articles from 30, 524 titles, 22 populations from 21 reports were included. In 9 cohorts, there were consistent, adjusted associations between baseline systolic BP and progression of arterial stiffness (11 781 patients; standardized β=0.041; 95% CI, 0.026–0.055; *P*<0.001; *P* value for heterogeneity=0.70), equivalent to a 1.14‐m/s increase in standard carotid‐femoral pulse wave velocity per decade per 20–mm Hg systolic BP, independent of age. Unstandardized, adjusted associations were similar (1762 patients; β=0.0047; 95% CI, 0.004–0.006; *P*<0.001; *P* value for heterogeneity=0.64), equivalent to a 0.94‐m/s increase per decade per 20–mm Hg systolic BP. In limited studies, standardized associations between mean BP and arterial stiffness progression were not significant and heterogeneous (913 patients; β=0.039; 95% CI, −0.008 to 0.086; *P*=0.11; *P* value for heterogeneity=0.03).

**Conclusions:**

Baseline systolic BP was associated with a clinically important progression of arterial stiffness, independent of age, providing a reference for the potential effect of arterial stiffness in mediating changes in clinical outcomes associated with hypertension and providing a reference value to aid clinical trial design.

**Nonstandard Abbreviations and Acronyms**
BP: blood pressure
cfPWV: carotid‐femoral pulse wave velocity
DBP: diastolic blood pressure
MBP: mean blood pressure
PWV: pulse wave velocity
SBP: systolic blood pressure

**Clinical Perspective**

**What Is New?**

- In this meta‐analysis, there was an estimated standardized regression coefficient of 0.041 between baseline systolic blood pressure and progression of aortic stiffness, which translates to a 1.14‐m/s increase per decade in standard carotid‐femoral pulse wave velocity for every 20–mm Hg systolic blood pressure.

**What Are the Clinical Implications?**

- Clinical harms caused by hypertension may partly be mediated by progression of arterial stiffness over time, independent of age, sex, and cardiovascular risk factors, a relationship that is large enough to be clinically significant over time.
- Reducing hypertension early has the potential to prevent progression of later‐life arterial stiffness and therefore associated clinical sequelae, and this relationship should be considered in clinical practice and clinical trial design.

Arterial stiffness is a robust predictor of cardiovascular events and mortality, independent of other risk factors, with a 1‐m/s increase in carotid‐femoral pulse wave velocity (cfPWV) associated with an increased risk of cardiovascular events, cardiovascular mortality, and all‐cause mortality by 14%, 15%, and 15%, respectively.^1^ The renal and cerebral circulations are particularly vulnerable to the effects of increased arterial stiffness because of transmission of increasingly pulsatile blood pressure (BP) to low‐resistance vascular beds.^2^ This results in a strong association between aortic stiffness, arterial pulsatility, and cerebral small vessel disease, which is implicated in up to 30% of strokes and 40% of dementia, as well as damage to the renal microcirculation, leading to renal insufficiency.^3^, ^4^, ^5^ Effects of hypertension and age are synergistic, resulting in a doubling of the risk of stroke for every decade past 55 years of age, whereas chronic kidney disease prevalence doubles from 65 to 74 years of age versus >75 years of age.^6^, ^7^

Arterial stiffness is most strongly associated with age and hypertension in cross‐sectional studies,^8^, ^9^ with weaker associations with inflammatory illnesses^10^ and diabetes mellitus.^11^ There are well‐defined reference values across the population for arterial stiffness^12^ and a clear consensus as to measurement methods enabling application to clinical practice.^13^ However, despite studies reporting associations between hypertension and progression of arterial stiffness, the magnitude of this relationship and the degree to which antihypertensive treatment may prevent progression of arterial stiffness and resulting clinical events are poorly defined. In addition, it is unclear whether arterial stiffness is a cause or a consequence of hypertension or whether both processes are mutually reinforcing.^14^ A more precise estimate of the magnitude of association between hypertension and progression of arterial stiffness will allow better understanding of the potential role of arterial stiffness in mediating the effect of hypertension on clinical events, the maximum possible magnitude of the effect of antihypertensives, and can therefore support realistic power calculations required for clinical trials.

Therefore, we performed a meta‐analysis of the magnitude of association between elevated BP and progression of arterial stiffness.

## Methods

### Search Strategy

EMBASE and Medline were searched between inception and May 24, 2019, to identify longitudinal studies reporting an association between BP and progression of arterial stiffening (Data S1). Accepted outcome measures included cfPWV, brachial‐ankle pulse wave velocity (PWV), and cardio‐ankle vascular index. Study titles, abstracts, and full‐text articles were reviewed sequentially (J.W.), with all included studies independently reviewed by 2 reviewers (J.W., A.W.; Table S1). All included studies were assessed for quality using the National Institutes of Health study quality assessment tool.^15^ Publication bias was assessed using funnel plots.^16^ The data that support the findings of this study are available from the corresponding author on reasonable request, and are all available in published journals.

### Data Extraction

Extracted primary effects included associations from general linear models between baseline BP (systolic BP [SBP], diastolic BP [DBP], or mean BP [MBP]) and progression of arterial stiffness, including both standardized and unstandardized β coefficients, either unadjusted or adjusted for demographic covariates and/or baseline arterial stiffness. Other extracted variables included demographics of the included population (age, sex, BP, arterial stiffness, and comorbidities), study characteristics (prospective versus retrospective and cohort versus case control versus trial), inclusion/exclusion criteria, length of follow‐up, loss to follow‐up, conclusions, method and frequency of stiffness measurement, and details of analytical models (model type, univariate versus multivariate analysis, and covariates included). Measures of uncertainty of all variables were extracted where available, including SD, SEM, or interquartile range.

Where only unstandardized β coefficients were reported, standardized β coefficients were calculated by multiplication of the unstandardized β coefficient by the ratio of the reported SD of the BP at baseline and the SD of change in arterial stiffness, with the opposite transformation for converting standardized β coefficients to unstandardized coefficients. Unstandardized β coefficients were transformed to the standard cfPWV, as defined by 80% of the distance between the carotid and femoral measurement site,^17^, ^18^ with transformation by standard formulae where possible or by the mean percentage difference to aortic length measured on magnetic resonance imaging.^18^ Where necessary, measures of uncertainty were estimated according to the Cochrane method, including SEM ($\text{SD}=\text{SE}\times \sqrt{n}$), interquartile range (SD=interquartile range/1.35), or, for the change in arterial stiffness, from the SD of arterial stiffness at baseline and follow‐up (${\text{SD}}_{\Delta }=\sqrt{\left({\text{SD}}_{1}^{2}+{\text{SD}}_{2}^{2}\right)}$), always taking the more conservative method of estimating the SD. Where studies reported population characteristics by subgroups, weighted means between subgroups were used to estimate mean values (eg, age) for the population as a whole. *P* values reported in this review are derived from the estimated CIs, so they may differ from those reported in the original articles.

### Statistical Analysis

Regression coefficients from unadjusted and adjusted analyses were combined by fixed and random effects meta‐analysis, weighted by the inverse variance.^19^ Unstandardized β coefficients were combined only for studies reporting the same method of measurement of BP and arterial stiffness, whereas standardized coefficients were also combined between studies reporting different measures of arterial stiffness, with sensitivity analyses restricted to studies reporting the same measures. Estimates of the clinical relevance of the effect size were determined per 20–mm Hg SBP per decade from unstandardized summary estimates. For standardized summary estimates, the effect size was transformed to an unstandardized summary effect size using estimated average values for the SD of baseline SBP and SD of change in PWV, derived from weighted averages (by study size) of the variance in all studies where this was reported.

Where the inverse variance for a β coefficient could not be estimated from reported information, an inverse variance was estimated from the ratio of the study size to the size of all studies in which the inverse variance was known, multiplied by the sum of the product of the inverse variance and study size for each study in which the inverse variance was known (inverse variance_j_=(n_j_/Σn)×(Σinverse variance_i…n_×n_i…n_)). Sensitivity analyses were performed without imputation and following adjustment for publication bias by trim and fill of smaller studies >1 SD from the summary estimate. Heterogeneity was assessed by I^2^ statistics and χ^2^ tests for heterogeneity. The meta‐analysis protocol was published on PROSPERO (CRD42019142440, International Prospective Register of Systematic Reviews) before data extraction, with subsequent focus on BP alone because of insufficient reporting of other modifiable risk factors from the same studies.

## Results

A total of 30 524 titles were identified and reviewed by title and abstract to determine eligibility. A total of 566 articles were reviewed in full, of which 21 (Figure S1) were eligible for inclusion in the meta‐analysis, with 1 study reporting data from 2 populations. There were no identified meta‐analyses of the effect of BP on progression of arterial stiffness. All included studies were prospective, with 15 of 21 studies reporting change in cfPWV, and 4 of 21 studies reporting brachial‐ankle PWV (Table S2), but studies varied significantly in size (51–8004 participants) and duration (6 months to 9.5 years). Most studies were of reasonable quality (Table S3), but there was a small risk of bias in some studies because of participant selection, blinding of outcome assessors to clinical characteristics, and dropout rate. There was moderate evidence of publication bias for SBP versus progression of arterial stiffness, with possible underreporting of negative associations (Figure S2), but resulting in limited impact on summary estimates.

**Figure 1:**
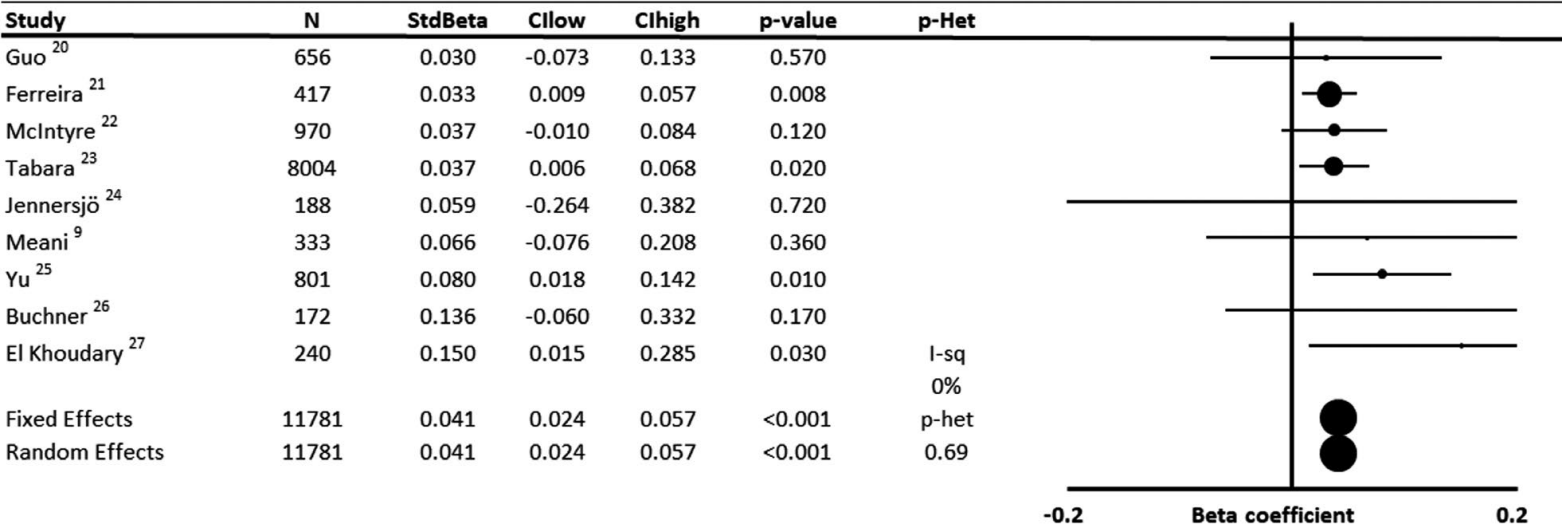
Forest plot of the effect of baseline systolic blood pressure on annual progression of arterial stiffness by meta‐analysis of standardized β regression coefficients from analyses adjusted for demographic variables. Effects were analyzed by both fixed and random effects meta‐analysis weighted by the inverse variance. CIhigh indicates CI upper limit; CIlow, CI lower limit; I‐sq, I^2^ statistic; N, number of subjects; *P*‐Het, *P* value for heterogeneity; and StdBeta, standardized regression coefficient.

**Figure 2:**
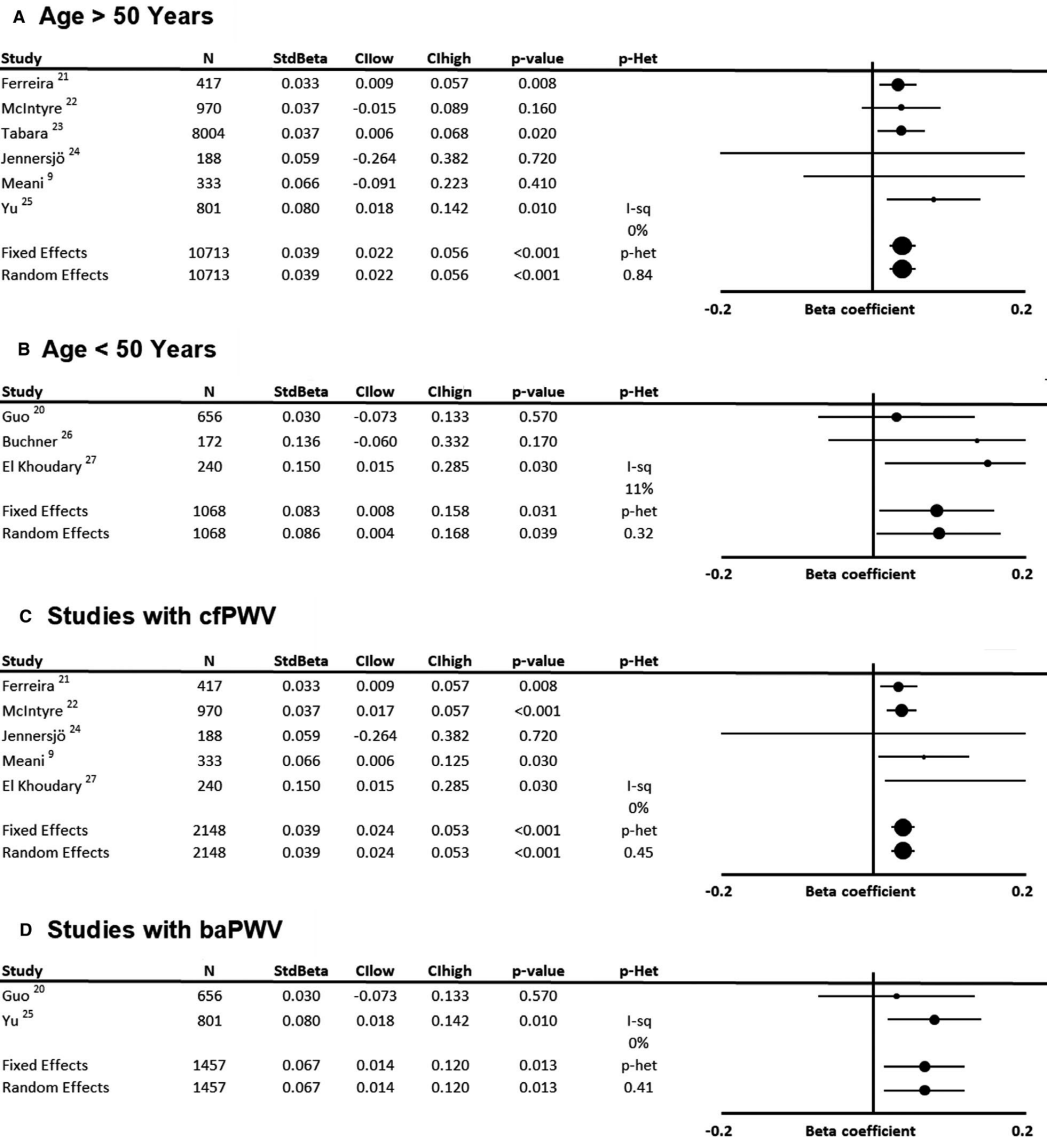
Forest plot of the effect of baseline systolic blood pressure on annual progression of arterial stiffness by meta‐analysis of standardized β regression coefficients from analyses adjusted for demographic variables and stratified by age. **A**, Mean age >50 years old. **B**, Mean age <50 years old. **C**, Including studies reporting effects on progression of carotid‐femoral pulse wave velocity (cfPWV). **D**, Including studies reporting effects on brachial‐ankle pulse wave velocity (baPWV). Effects were analyzed by both fixed and random effects meta‐analysis weighted by the inverse variance. CIhigh indicates CI upper limit; CIlow, CI lower limit; I‐sq, I^2^ statistic; N, number of subjects; *P*‐Het, *P* value for heterogeneity; and StdBeta, standardized regression coefficient.

In 9 populations including 11 781 participants, there was a consistent standardized association between baseline SBP and increase in arterial stiffness in adjusted linear models (Figure 1, ^9^, ^20^, ^21^, ^22^, ^23^, ^24^, ^25^, ^26^, ^27^; β=0.041; 95% CI, 0.24–0.057; *P*<0.001), with no significant heterogeneity between studies (*P* value for heterogeneity=0.69), including after exclusion of the study reporting carotid‐radial PWV (β=0.040; 95% CI, 0.024–0.057; *P*<0.001). This association was consistent for studies assessing cfPWV or brachial‐ankle PWV (Figure 2, ^9^, ^20^, ^21^, ^22^, ^23^, ^24^, ^25^, ^26^, ^27^, ^28^) and corresponds to an ≈1.14‐m/s increase in standard cfPWV^17^ per 20–mm Hg SBP per decade (following standardization of 1 study estimate), in addition to the effect of increasing age. There was a range of reported variances for change in PWV between studies, but systematic exclusion of single studies resulted in summary estimates within the CI of the overall mean (ranging from 0.97–1.32 m/s). Excluding studies where measures of uncertainty were imputed had no significant effect (10 478 participants; β=0.041; 95% CI, 0.023–0.059; *P*<0.001), and the effect was similar when excluding studies that also reported unstandardized coefficients (10 773 participants; β=0.042; 95% CI, 0.025–0.059; *P*<0.001).

In 5 studies reporting unadjusted associations between SBP and progression of arterial stiffness, there was a stronger association with progression of arterial stiffness (standardized β=0.063; 95% CI, −0.002 to 0.129; *P*=0.06; Figure S3A), reflecting covariance between age and SBP. However, there was significant heterogeneity (*P* value for heterogeneity=0.0062), particularly because of limited interventional studies reporting negative correlations. On restricting the analysis to noninterventional studies, there was no significant heterogeneity and a consistent positive association between SBP and progression of arterial stiffness (Figure S3B).

**Figure 3:**
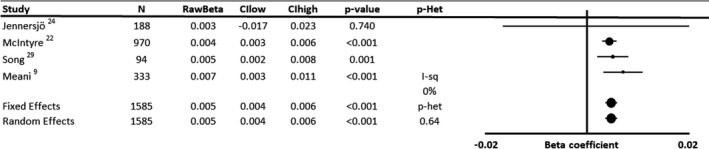
Forest plot of the effect of baseline systolic blood pressure on annual progression of arterial stiffness by meta‐analysis of unstandardized β regression coefficients from analyses adjusted for demographic variables. Effects were analyzed by both fixed and random effects meta‐analysis weighted by the inverse variance. CIhigh indicates CI upper limit; CIlow, CI lower limit; I‐sq, I^2^ statistic; N, number of subjects; *P*‐Het, *P* value for heterogeneity; and RawBeta, unstandardized regression coefficient.

There was a similar magnitude of effect in studies reporting unstandardized, adjusted β coefficients between SBP and progression of cfPWV (Figure 3, ^9^, ^22^, ^24^, ^29^; 1762 patients; 0.0047 m/s per year per 1–mm Hg SBP; 95% CI, 0.0035–0.0059; *P*<0.001), corresponding to 0.94‐m/s cfPWV per 20–mm Hg SBP per decade, with no significant heterogeneity. Associations were similar when excluding studies where measures of uncertainty were imputed (0.0049 m/s per year per 1–mm Hg SBP; 95% CI, 0.002–0.0079; *P*=0.001).

**Figure 4:**
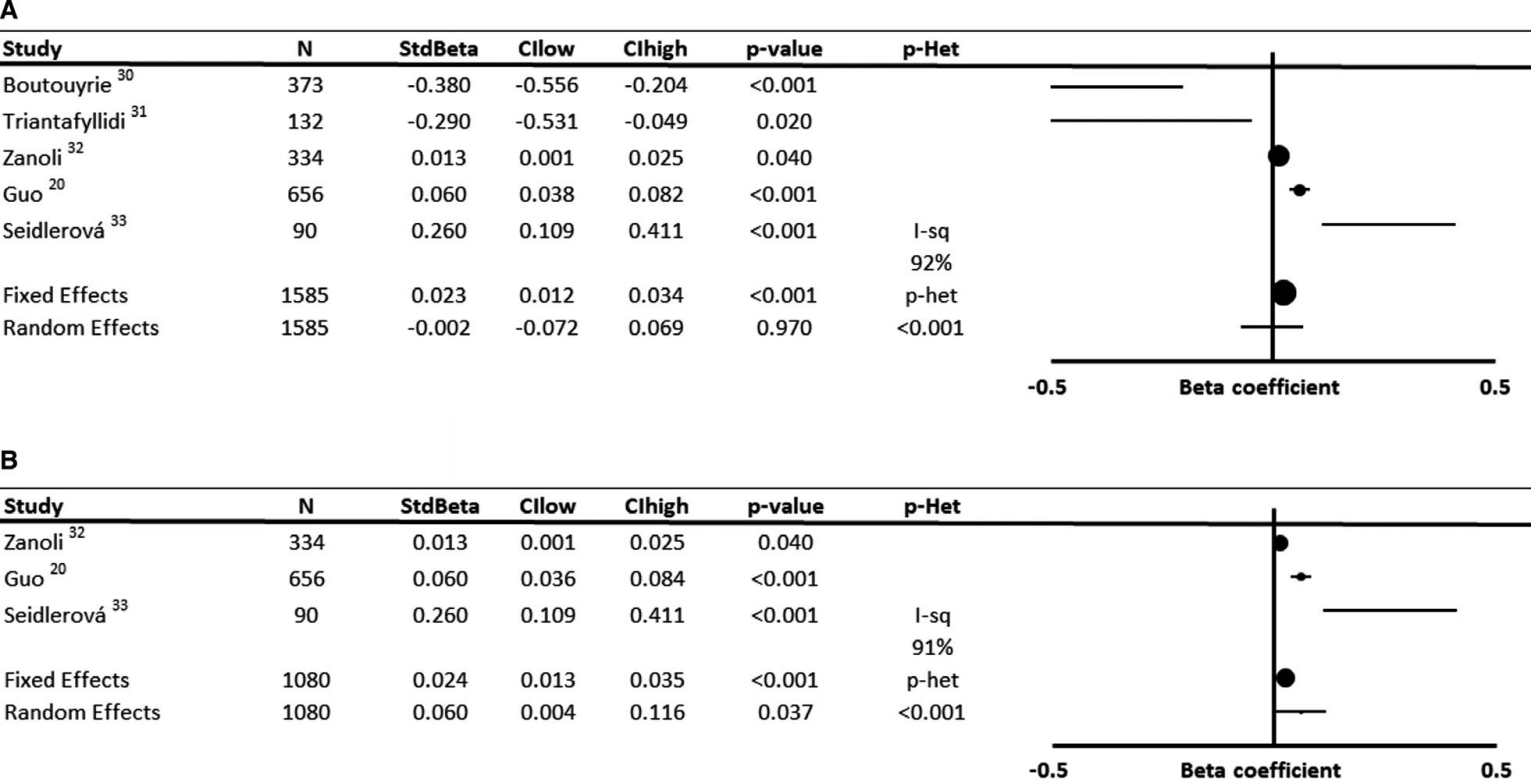
Forest plot of the effect of baseline mean blood pressure on annual progression of arterial stiffness by meta‐analysis of standardized β regression coefficients from analyses adjusted for demographic variables. Effects were analyzed by both fixed and random effects meta‐analysis weighted by the inverse variance. **A**, Includes all studies according to original inclusion criteria. **B**, Excludes interventional studies with a reported reduction in pulse wave velocity during follow‐up. CIhigh indicates CI upper limit; CIlow, CI lower limit; I‐sq, I^2^ statistic; N, number of subjects; *P*‐Het, *P* value for heterogeneity; and StdBeta, standardized regression coefficient.

**Figure 5:**
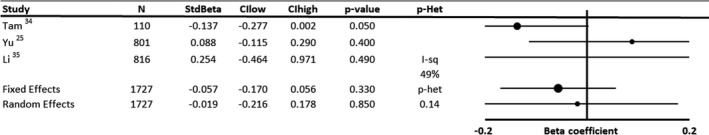
Forest plot of the effect of baseline diastolic blood pressure on annual progression of arterial stiffness by meta‐analysis of standardized β regression coefficients from analyses not adjusted for demographic variables. Effects were analyzed by both fixed and random effects meta‐analysis weighted by the inverse variance. CIhigh indicates CI upper limit; CIlow, CI lower limit; I‐sq, I^2^ statistic; N, number of subjects; *P*‐Het, *P* value for heterogeneity; and StdBeta, standardized regression coefficient.

There was no significant association between baseline MBP (Figure 4, ^20^, ^30^, ^31^, ^32^, ^33^) or DBP (Figure 5, ^25^, ^34^, ^35^) and progression of arterial stiffness, because of a limited number of available studies and significant heterogeneity. Of 5 studies reporting associations between MBP and progression of arterial stiffness, 2 were interventional studies with negative associations, compared with positive associations in prospective observational studies. However, even after exclusion of interventional studies, substantial uncertainty in the magnitude of association between MBP and progression of arterial stiffness remained (Figure 4, ^20^, ^30^, ^31^, ^32^, ^33^).

Compared with the association with baseline SBP, there was a stronger association between age and progression of arterial stiffness in fully adjusted models (Figure S4), although with significant heterogeneity (*P* value for heterogeneity<0.001) between studies reflecting variation in population characteristics and adjusted covariates. However, there was no statistically significant difference in the association between baseline SBP and progression of arterial stiffness when comparing studies with a mean age of <50 years compared with studies with a mean age of >50 years (Figure 2, ^9^, ^20^, ^21^, ^22^, ^23^, ^24^, ^25^, ^26^, ^27^, ^28^; β interaction=0.026; 95% CI, −0.025 to 0.0771; *P*=0.32), or for the interaction between mean age and the association between baseline SBP and change in PWV in a metagression (*P*=0.28). Furthermore, sensitivity analyses including only studies that adjusted for both baseline age and baseline arterial stiffness were highly consistent, with similar effect sizes to the primary meta‐analysis (Figures S5 and S6).

## Discussion

In this meta‐analysis, there was a consistent association between baseline SBP and progression of arterial stiffness, after adjustment for demographic measures, with a similar magnitude of effect in independent studies reporting standardized or unstandardized β coefficients. The effect size was clinically important, with an increase of 1.14 or 0.94 m/s in PWV per decade for every 20–mm Hg increase in SBP, after adjustment for the effect of age. However, reported associations with MBP or DBP were weaker and inconsistent because of the limited number of heterogeneous studies.

Arterial stiffness is one of the strongest markers of increased cardiovascular risk, independent of age, sex, and cardiovascular risk factors, and is associated in cross‐sectional and prospective studies with mortality, renal dysfunction, stroke, and dementia.^1^, ^5^, ^36^, ^37^ Current hypertension guidelines identify arterial stiffness as a marker of end organ damage,^38^, ^39^ reflecting its association with hypertension in cross‐sectional analysis,^40^ whereas several studies have reported longitudinal associations between baseline hypertension or SBP and progression of arterial stiffness.^28^, ^40^ However, these studies vary in the consistency and magnitude of this association, and vary in the method of assessment of PWV, both by device and in measurement of the carotid femoral differences for estimation of cfPWV. This requires transformation of raw PWV values to the standard index (80% of the direct carotid‐femoral distance), adding a potential source of uncertainty in the estimate. Overall, this meta‐analysis therefore provides the best available estimate of the temporal relationship between raised SBP and progression of arterial stiffness, independent of age and initial severity of stiffness. Furthermore, it confirms that the available reports of this association are remarkably consistent, that the association is statistically robust, and that it is of sufficient magnitude to potentially explain a clinically important proportion of the burden of cardiovascular disease conferred by elevated SBP. The ≈1‐m/s increase in standard cfPWV^17^ per decade per 20–mm Hg SBP would correlate with ≈15% excess mortality per decade from previous studies.^1^ This therefore provides an estimate of the potential reduction in cardiovascular events caused by an achieved reduction in SBP if mediated by a reduction in arterial stiffness, and an estimate of the maximal difference in change in cfPWV that could result from interventions to control cfPWV in trials and clinical practice. However, this estimate would need transformation by standard formula to equivalent reference values of alternative methods of measurement, such as brachial‐ankle PWV,^12^ and for methods of measuring PWV with alternative devices and methods of estimating the aortic length.^18^

SBP, which is more strongly dependent on the pulsatile component of BP than DBP, was more strongly associated with longitudinal progression of arterial stiffness than markers of the constant component of BP (MBP). As such, pulsatile blood flow may be more important in driving increases in arterial stiffness caused by either a greater intermittent maximum pressure or a greater shear stress, rather than the total BP burden. Unfortunately, studies did not report associations with pulse pressure compared with mean pressure to assess this more directly. Also, despite the temporal order of the relationship, it is still possible that these associations are caused by reverse causation, with elevated arterial stiffness inducing a decreased DBP and increased SBP, reflecting an increase in pulse pressure (pulse pressure=stroke volume×stiffness/arterial volume) attributable to increased reflection of the cardiac pulse wave from the peripheral circulation.^12^ Furthermore, the longitudinal relationship between arterial stiffness, midlife diastolic hypertension, and late‐life systolic hypertension is complex, with a modulation of the association with increasing age.^41^ Therefore, the identified associations may reflect confounding by elevated MBP or DBP at younger ages, but the available data were not sufficient to determine if the relationship between SBP and progression of arterial stiffness differs by age group. Finally, this meta‐analysis also confirms that despite the importance of baseline SBP, there is still a significantly stronger standardized association between age and progression of arterial stiffness, with approximately a 2.5‐ to 4‐fold greater effect size.

This review has some limitations. First, there was limited quantitative data available for both MBP and DBP, limiting the strength of conclusions as to the relative effect of the pulsatile (SBP) and the constant (MBP/DBP) components of BP, although the strength of association with SBP compared with available MBP data suggests that a significantly greater association with MBP is unlikely. Second, there was a large variation between studies in reporting of method of measurement, population characteristics, and adjustment for confounding variables. However, there was little heterogeneity between studies in estimates of the standardized β coefficient, after adjustment for age in particular. Third, studies varied in reporting of estimates of uncertainty. As such, these measures were conservatively estimated or imputed from available information, resulting in a potential underestimate of the precision of the meta‐analysis. However, sensitivity analyses in the more completely reported studies showed similar effect sizes. Fourth, there was a limited number of studies for each outcome measure, preventing stratification of analyses to identify interactions with SBP by age and sex.^21^ Fifth, all included studies used general linear models with change in PWV as the outcome. This, therefore, may not sufficiently allow for repeated measures or the collinearity between SBP and PWV at baseline, but this is unlikely to affect the conclusions given the consistency of the results between studies and consistency with limited reports from mixed‐effect longitudinal linear models.^42^ Finally, despite the low heterogeneity, there was still a large range of effect sizes reported, implying variation between populations. However, the mean estimate of the standardized relationship obtained from a random effects meta‐analysis was consistent with all individual studies and was biologically plausible.

This study provides a reference value for further studies to determine the magnitude of the effect of hypertension on progression of arterial stiffness and therefore the maximum potential effect of antihypertensive treatment mediated by BP reduction. This supports calculation of the power required for clinical trials to prevent progression of arterial stiffness where previous estimates of the direct effect of interventions are not available, both for antihypertensive medications and for other treatments. In addition, it provides a reference value to compare the relative effect of different classes of antihypertensive medications on arterial stiffness, and whether the demonstrated effect is as could be expected for the achieved reduction in SBP or whether non–BP‐mediated effects may be important. However, further studies are required to determine whether the estimated effect size is consistent between different demographic groups and provide a better estimate of the association with MBP or DBP and progression of arterial stiffness.

## Conclusions

This study provides the best estimate of the relationship between elevated SBP and progression of arterial stiffness, demonstrating consistent associations across independent studies, equating to approximately a 1‐m/s increase in PWV per 20–mm Hg SBP per decade. This is of sufficient magnitude to be associated with approximately a 15% greater relative mortality per decade. It therefore provides a reference to understand the potential role of arterial stiffness in mediating the clinical effects of hypertension, as well as the potential long‐term benefits of blood pressure lowering on clinical events through control of arterial stiffening, in addition to the impact of age. Finally, as the most accurate estimate available, it provides an expected effect size for determining sample sizes for future trials of agents to reduce progression of arterial stiffness. However, although this association may imply a greater importance of pulsatile hemodynamics in determining progression of arterial stiffness than the constant components of BP, further research is required to accurately determine the association with MBP and DBP, to reliably exclude an age‐dependent modification of these associations and exclude the possibility of reverse causation.

## Sources of Funding

This study received no specific funding. Dr Webb is funded by a Wellcome Trust Clinical Research Career Development Fellowship (206589/Z/17/Z) and a British Foundation Project grant (PG/16/38/32080).

## Disclosures

None.

## Supporting information


**Data S1**
**Tables S1–S3**
**Figures S1–S6**
**References** **43**, **44**
