# Supplementary material for: Systolic Blood Pressure and Longitudinal Progression of Arterial Stiffness: A Quantitative Meta‐Analysis
Source: J Am Heart Assoc. 2020 Aug 28;9(17):e017804. doi: 10.1161/JAHA.120.017804 (PMC7660776; doi:10.1161/JAHA.120.017804)
Supplement: Supplementary file 1 — Data S1 Tables S1–S3 Figures S1–S6 References 43 , 44 [file JAH3-9-e017804-s001.pdf]

SUPPLEMENTAL MATERIAL

Data S1.

Search Strategy:

Pubmed and EMBASE were searched with the following terms:

(("arterial stiff*" OR "arterial elasticity" OR "arterial compliance" OR "arterial distensibility" OR "arterial calcification" OR "vascular stiff*" OR "vascular elasticity" OR "vascular compliance" OR "vascular distensibility" OR "vascular calcification" OR "carotid stiff*" OR "carotid elasticity" OR "carotid compliance" OR "carotid distensibility" OR "carotid calcification" OR "aortic stiff*" OR "aortic elasticity" OR "aortic compliance" OR "aortic distensibility" OR "aortic calcification" OR "pulse wave velocity" OR "pulse wave transit time" OR "pulse transit time" OR "cfPWV" OR "baPWV" OR "PWV" OR "cardio-ankle vascular index" OR "arterial stiffness index" OR "HASI" OR "AASI" OR "ABPI" OR "CAVI" OR "ankle-brachial pressure index" OR "ankle-brachial index" OR "modulus of elasticity" OR "young's modulus" OR "elastic modulus" OR "pulse wave analysis" OR "augmentation index" OR "PWA" OR "AIx" OR "pulse pressure") AND ("randomi?ed control trial" OR "cohort" OR "ecological stud*" OR "longitudinal" OR "follow-up" OR "randomised control* trial" OR "RCT" OR "health database" OR "case-control" OR "prospective" OR "population-based" OR "clinical trial" OR "long-term" OR "progression"))

Table S1. Inclusion and Exclusion criteria.

Inclusion	Exclusion
Study in humans	Animal studies
Longitudinal studies including cohorts, RCTs, intervention and case-control study designs	Cross-sectional analyses
Study in adults of any sex/ethnicity	Studies in children/adolescents
Studies written in English	Participants with conditions directly impacting aortic function (including but not limited to):<br>- Marfan's, coarctation of the aorta<br>- Conditions with severe systemic physiological effects likely to lead to atypical aortic changes (e.g. ESRD, systemic sclerosis)
Reports numerical association between modifiable risk factor and change in arterial stiffness over time.	Pregnant women
Follow up duration ≥6 months/24 weeks	Quantitative change in stiffness not recorded
	Case series
	Exclusively studying participants with CKD≥4 or on dialysis
	Randomised controlled trials only reporting treatment effects

Table S2. Characteristics of studies included in the meta-analysis.

Study	N	Follow up (years)	Age (years)	SD	Design	% Male	Measure	Device	Distance method
Tabara et al.[23]	8004	5	53.1	13.3	Cohort	32.4	CAVI	Vasera-1500	From height
McIntyre et al.[22]	970	5	70	8.1	Cohort	39	cfPWV	Vicorder	SSN – FA
Meani et al.[9]	333	3.75 +-.53	54.5	12.6	Cohort	56.7	cfPWV	Complior	(CA – FA) * 0.8
Yu et al.[25]	801	up to 7	50.8	12.9	Case-control	43.3	baPWV	MB3000	(ht–ank) – (ht–arm)
Guo et al.[20]	656	5.7 (4-7.9)	45.2	2.9	Cohort	100	baPWV	VP2000	From height
Jennersjö et al.[24]	188	4	60.4	3.3	Cohort	72	cfPWV	Sphygmocor	(SSN–FA) – (SSN–CA)
Ferreira et al.[21]	417	4.2+-0.6	60.4	9.5	Cohort	35.7	cfPWV	Complior	(CA – FA) * 0.8
Song et al.[29]	271	4	53.78	6.64	Interventional	50	cfPWV	CMR	Direct visualisation
AlGhatrif et al.[42]	775	9.3+-6	59	15.7	Cohort	45.4	cfPWV	Comp / Sphyg	(SSN-Um-FA)–(SSN-CA)
El Khoudary et al.[27]	240	4.6+-0.2	45	2.9	Cohort	100	cfPWV	VP2000	(SSN-Um-FA)–(SSN-CA)
Buchner et al.[26]	172	0.5	48.8	14.6	Interventional	54.5	crPWV	Sphygmocor	(SN-radial) – (SN-CA)
Zanoli et al.[32]	167	4 (2.5-5.7)	38	21	Case-control	57	cfPWV	Sphygmocor	(CA – FA) * 0.8
Triantafyllidi et al.[31]	132	2.95+-0.7	55	11	Interventional	53	cfPWV	Complior	FA-CA
Seidlerová et al.[33]	90	9.5 (8.5-9.7)	66.9	5.1	Cohort	20	cfPWV	Sphygmocor	(SSN–FA) – (SSN–CA)
Takaki et al.[43]	50	1	60.9	7	RCT	80	baPWV	AT-form	-
Maia-Leite et al.[44]	91	7.6+-2	47.9	8.1	Cohort	91.7	cfPWV	Complior	(CA – FA) * 0.8
Boutouyrie et al.[30]	373	2	56.4	14.7	RCT	53.6	cfPWV	Sphygmocor	(CA – FA) * 0.8
Karatzi et al.[45]	115	5	47.8	9.1	Cohort	60.9	cfPWV	Complior	(CA – FA) * 0.8
Tam et al.[34]	110	1	53	13	RCT	23	baPWV	VP2000	-
Li et al.[35]	816	5	39.1	7.8	Cohort	58.3	cfPWV	Sphygmocor	(SSN–FA) – (SSN–CA)
Ohyama et al.[46]	1395	10	60	9	Cohort	47	aPWV	CMR	Direct visualisation

Study characteristics and method of assessment of arterial stiffness are reported, including by device and method of estimation of aortic length. PWV=pulse wave velocity; CAVI=cardio-ankle vascular index; cfPWV=carotid-femoral; ba=brachial-ankle; cr= carotid-radial; SSN = suprasternal notch; SN=sternal notch; FA= femoral artery; CA = carotid artery.

Table S3. Measures of study quality which differed between studies; Y=yes, N=No, NR = Not reported.

Study	Participation >50%	Adequate outcome assessor blinding	Dropout rate <20%
Tabara[23]	Y	N	Y
McIntyre[22]	Y	N	Y
Meani[9]	Y	N	Y
Yu [25]	Y	Y	Y
Guo[20]	Y	N	N
Jennersjö[24]	N	N	N
Ferreira[21]	Y	Y	NR
Song[29]	Y	N	Y
AlGhatrif[42]	Y	N	Y
El Khoudary[27]	Y	N	N
Buchner[26]	NR	N	Y
Zanoli[32]	NR	Y	NR
Triantafyllidi[31]	Y	N	Y
Seidlerová[33]	Y	N	N
Takaki[43]	NR	Y	Y
Maia-Leite[44]	Y	N	N
Boutouyrie[30]	NR	Y	Y
Karatzi[45]	Y	N	Y
Tam[34]	Y	Y	Y
Li[35]	Y	N	N
Ohyama[46]	NR	NR	NR

Figure S1. PRISMA Flowchart.

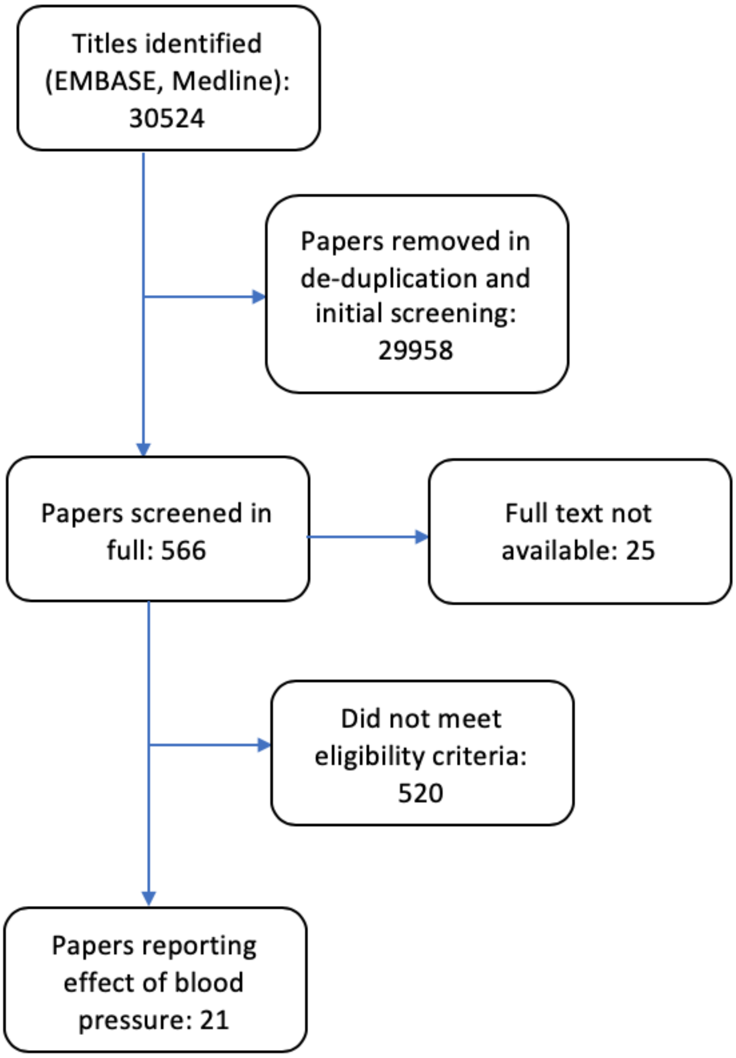

Figure S2. Assessment of publication bias.

A)

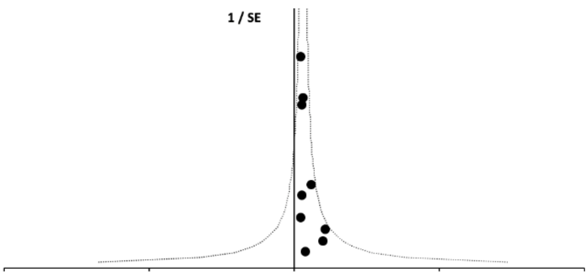

B)

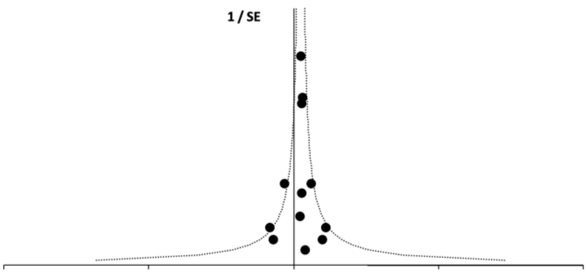

C)

Study	N	StdBeta	Cilow	Cihigh	p-value	p-Het
Sensitivity analysis 3 et al.	240	-0.114	-0.249	0.021	0.100	
Sensitivity analysis 2 et al.	172	-0.100	-0.296	0.096	0.320	
Sensitivity analysis 1 et al.	801	-0.044	-0.106	0.018	0.160	
Guo et al.	656	0.030	-0.073	0.133	0.570	
Ferreira et al.	417	0.033	0.009	0.057	0.007	
McIntyre et al.	970	0.037	-0.033	0.107	0.300	
Tabara et al.	8004	0.037	0.006	0.068	0.020	
Meani et al.	333	0.040	0.010	0.070	0.009	
Jennersjö et al.	188	0.054	-0.269	0.377	0.740	
Yu et al.	801	0.080	0.018	0.142	0.010	
Buchner et al.	172	0.136	-0.060	0.332	0.170	
El Khoudary et al.	240	0.150	0.015	0.285	0.030	I-sq
						41%
Fixed Effects	12994	0.033	0.019	0.048	<0.001	p-het
Random Effects	12994	0.031	0.007	0.055	0.010	0.07

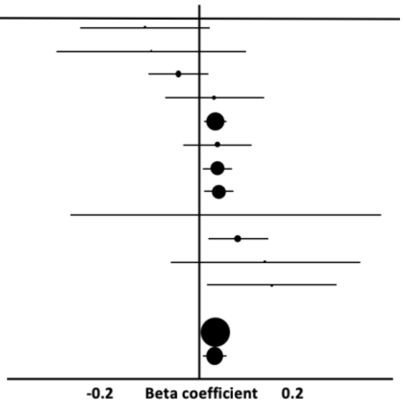

A: Funnel plot for the studies reporting adjusted standardised beta-coefficients for the relationship between baseline SBP and progression of arterial stiffness. B: Funnel plot for the studies reporting adjusted standardised beta-coefficients for the relationship between baseline SBP and progression of arterial stiffness, with imputed values for studies greater than 1 SD above the summary value, reflected about 0.036 to estimate effects of possible publication bias. 1/SE = 1/standard error. C: Meta-analysis of standardised, adjusted beta-coefficients for baseline SBP versus progression of arterial stiffness, including imputed values.

Figure S3. Forest plots showing the magnitude of unadjusted associations between baseline SBP and progression of arterial stiffness.

A

Study	N	StdBeta	CIlow	CIhigh	p-value	p-Het
Tam [34]	110	-0.165	-0.305	-0.025	0.020	
El Khoudary [27]	240	0.100	-0.018	0.218	0.100	
Li [35]	816	0.101	-0.042	0.243	0.170	
Yu [25]	801	0.177	0.045	0.309	0.009	
Karatzi [45]	115	0.328	-0.318	0.974	0.320	I-sq
						72%
Fixed Effects	2082	0.063	-0.002	0.129	0.060	p-het
Random Effects	2082	0.065	-0.071	0.202	0.350	0.0062

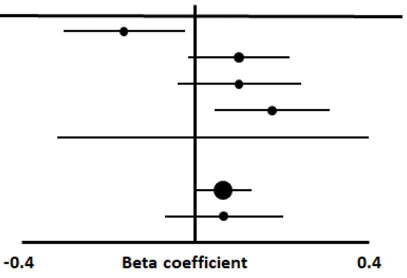

B

Study	N	StdBeta	CIlow	CIhigh	p-value	p-Het
El Khoudary [27]	240	0.100	-0.018	0.218	0.100	
Li [35]	816	0.101	-0.042	0.243	0.170	
Yu [25]	801	0.177	-0.007	0.361	0.060	
Karatzi [45]	115	0.328	-0.571	1.227	0.470	I-sq
						0%
Fixed Effects	1972	0.117	0.036	0.198	0.005	p-het
Random Effects	1972	0.117	0.036	0.198	0.005	0.86

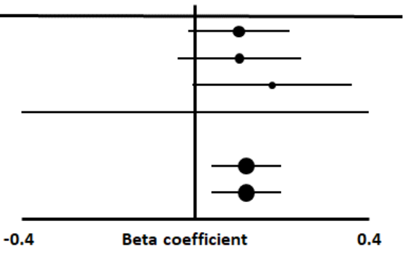

A: Unadjusted relationships between baseline SBP and progression of arterial stiffness. B: Unadjusted relationships between baseline SBP and progression of arterial stiffness, excluding interventional studies. Effects were analysed by both fixed and random effects meta-analysis weighted by the inverse variance. StdBeta – Standardised regression coefficient; Cilow – confidence interval lower limit; Cihigh – confidence interval upper limit; p-Het – p-value for heterogeneity; N – number of subjects; I-sq – I^2 statistic.

Figure S4. Forest plot of the effect of baseline age on annual progression of arterial stiffness by meta-analysis of standardised beta regression coefficients from analyses, adjusted for demographic variables.

Study	N	StdBeta	CIlow	CIhigh	p-value	p-Het
Maia-Leite [44]	91	0.030	0.005	0.055	0.020	
Jennersjö [24]	188	0.065	0.044	0.086	<0.001	
Meani [9]	333	0.089	0.049	0.130	<0.001	
McIntyre [22]	970	0.148	0.108	0.187	<0.001	
Zanoli [32]	334	0.150	0.051	0.249	0.003	
Ferreira [21]	417	0.367	0.112	0.622	0.005	
Tabara [23]	8004	0.508	0.461	0.555	<0.001	I-sq
						98%
Fixed Effects	10337	0.105	0.092	0.119	<0.001	p-het
Random Effects	10337	0.182	0.069	0.295	0.002	<0.001

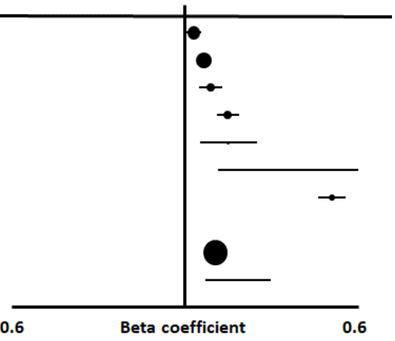

Effects were analysed by both fixed and random effects meta-analysis weighted by the inverse variance. StdBeta – Standardised regression coefficient; Clow – confidence interval lower limit; Chigh – confidence interval upper limit; p-Het – p-value for heterogeneity; N – number of subjects; I-sq – I^2 statistic

Figure S5. Associations between baseline SBP and progression of arterial stiffness, only including studies reporting associations adjusted for baseline age.

Study	N	StdBeta	CIlow	CIhigh	p-value	p-Het
Guo [20]	656	0.030	-0.073	0.133	0.570	
Ferreira [21]	417	0.033	0.009	0.057	0.008	
Tabara [23]	8004	0.037	0.006	0.068	0.020	
Benetos [28]	483	0.052	-0.011	0.115	0.100	
Jennersjö [24]	188	0.054	-0.269	0.377	0.740	
Meani [9]	333	0.066	-0.086	0.218	0.390	
Buchner [26]	172	0.136	-0.060	0.332	0.170	
El Khoudary [27]	240	0.150	0.015	0.285	0.030	I-sq
						0%
Fixed Effects	10493	0.039	0.021	0.056	<0.001	p-het
Random Effects	10493	0.039	0.021	0.056	<0.001	0.77

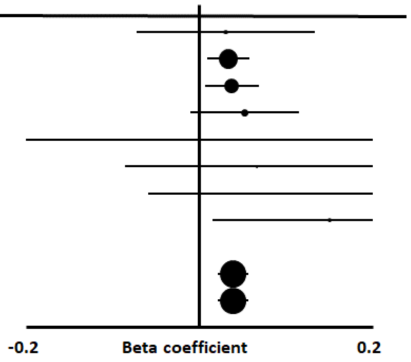

Effects were analysed by both fixed and random effects meta-analysis weighted by the inverse variance. StdBeta – Standardised regression coefficient; Cilow – confidence interval lower limit; Cihigh – confidence interval upper limit; p-Het – p-value for heterogeneity; N – number of subjects; I-sq – I^2 statistic.

Figure S6. Associations between baseline SBP and progression of arterial stiffness, including studies reporting associations adjusted for baseline age and baseline arterial stiffness.

Study	N	StdBeta	CIlow	CIhigh	p-value	p-Het
Guo [20]	656	0.030	-0.073	0.133	0.570	
Ferreira [21]	417	0.033	0.009	0.057	0.008	
Tabara [23]	8004	0.037	0.006	0.068	0.020	
Jennersjö [24]	188	0.054	-0.269	0.377	0.740	I-sq
						0%
Fixed Effects	9265	0.034	0.016	0.053	<0.001	p-het
Random Effects	9265	0.034	0.016	0.053	<0.001	1

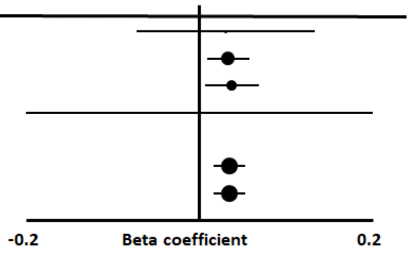

Effects were analysed by both fixed and random effects meta-analysis weighted by the inverse variance. StdBeta – Standardised regression coefficient; Cilow – confidence interval lower limit; Cihigh – confidence interval upper limit; p-Het – p-value for heterogeneity; N – number of subjects; I-sq – I^2 statistic.
